# Non‐invasive prenatal screening for fetal triploidy using single nucleotide polymorphism‐based testing: Differential diagnosis and clinical management in cases showing an extra haplotype

**DOI:** 10.1002/pd.6169

**Published:** 2022-05-21

**Authors:** Valerie Kantor, Russ Jelsema, Wenbo Xu, Wendy DiNonno, Kathryn Young, Zachary Demko, Peter Benn

**Affiliations:** ^1^ Natera, Inc. San Carlos California USA; ^2^ Department of Genetics and Genome Sciences UConn Health Farmington Connecticut USA

## Abstract

**Objective:**

An extra haplotype is infrequently encountered in single nucleotide polymorphism(SNP)‐based non‐invasive prenatal testing (NIPT) and is usually attributed to an undetected twin or triploidy. We reviewed a large series to establish relative frequencies of these outcomes and identify alternative causes.

**Methods:**

In 515,804 women receiving NIPT from September 2017 through March 2019, all results with an extra haplotype were reviewed. Known viable and vanished twin pregnancies were excluded. For positive cases, pregnancy outcome information was sought.

**Results:**

Of 1005 results with an extra haplotype (1 in 513), pregnancy outcome was available for 773 cases: 11% were confirmed or suspected triploidy; 65% to vanished twin; 10% with pregnancy loss. Rare explanations included complete mole, chimera, undisclosed donor egg pregnancy, maternal organ transplant and one instance of maternal neoplasm. Among triploid cases that were detected and independently confirmed, 23/27 (85%) were diandric.

**Conclusion:**

SNP‐based NIPT, with detection of an extra haplotype, is 11% predictive of triploidy. For results with an extra haplotype, ultrasound is recommended to establish viability, evaluate for twins (viable or vanished), and detect findings consistent with triploidy. Review of patient history, serum screening, and ultrasound will reduce the number of CVS or amniocenteses necessary to confirm a diagnosis of triploidy.

## Key points

What's already known about this topic?

- Both digynic and diandric triploidy are occasionally seen in pregnancies but are extremely rare in live‐births
- In cell‐free DNA prenatal screening, the presence of an extra haplotype could be indicative of either twin pregnancy or triploidy

What does this study add?

- Outcomes for a large series of prenatal screening tests where an extra haplotype was identified in cell‐free DNA
- Complete moles, chimeras, transplantation, and donor egg are additional explanations for extra haplotypes
- We discuss prenatal management when an extra haplotype is observed in cell‐free DNA

## 1 INTRODUCTION

Triploidy is defined as the presence of three copies of each chromosome in a cell, instead of the usual two sets of homologs. In triploid fetuses, the extra set of chromosomes can be maternal (digynic) or paternal (diandric) in origin. On ultrasound examination, digynic triploidy is typically associated with severe fetal growth restriction and a small non‐cystic placenta. In contrast, diandric triploidy often demonstrates normal fetal growth with multiple placental cysts.^1^, ^2^ For pregnancies with a diagnosis of triploidy, serial human chorionic gonadotropin (HCG) tests are recommended to ensure complete removal of residual trophoblastic tissue.^3^, ^4^ Ultrasound visualized fetal abnormalities in triploidy include open neural tube defects, ventral wall defects, syndactyly, and other anatomic abnormalities.^5^ The incidence of triploidy at approximately 11–13 weeks gestation has been estimated to be approximately 1 in 4800 pregnancies (Supplemental Table 1), decreasing to <1:27,000 in the second trimester.^6^ Rare cases (possibly mosaic or chimeric) can survive to the third trimester, and these usually result in stillbirth or neonatal death.^7^ Although reports exist regarding multiple recurrences of digynic triploid pregnancies,^8^ the overall risk for recurrence has not been established. For diandric triploid pregnancies, recurrence risk is approximately twice the general population risk.^9^

Because of the risk for maternal malignant trophoblastic disease, early detection of triploidy is advantageous.^10^ Conventional first and second trimester maternal serum biochemical tests can help screen for triploid pregnancies,^11^, ^12^ but in some countries serum testing is less widely utilized because of the increasing use of cell‐free DNA (cfDNA) based non‐invasive prenatal testing (NIPT) for autosomal trisomies, sex chromosome abnormalities and some other imbalances. Counting‐based NIPT methods are unable to routinely detect triploidy because there is no proportional change in the number of DNA fragments across the different autosomes. As ACOG noted in a 2020 Practice Bulletin,^13^ SNP‐based NIPT can identify triploidy by detecting the presence of the additional haploid chromosome set.^14^ However, the SNP pattern seen with triploidy can sometimes be difficult to distinguish from the pattern seen with dizygotic twins, particularly when the fetal fraction is low.^15^

Curnow et al.^14^ reported the outcomes for an initial cohort of 76 instances where there was SNP‐based evidence for an extra haplotype in cfDNA. Curnow et al., also provided initial estimates for the proportions of these results attributable to vanished twins, viable twins, or triploidy. Since Curnow et al.'s report, pregnancies with two viable fetuses can be analyzed using a SNP‐based NIPT. In this report, we present follow‐up information for 773 pregnancies with extra haplotypes, collected since the introduction of twin NIPT. We also provide additional causes for the presence of extra haplotypes in cell‐free DNA.

## 2 METHODS

We reviewed the laboratory results for all women in the United States who had SNP‐based NIPT performed at Natera, Inc., from September 2017 through March 2019. To meet research compliance requirements, the study was limited to testing performed for women residing in the United States. The test methodology and algorithms to assess aneuploidy in singleton and twin pregnancies have been described.^16^, ^17^, ^18^, ^19^ Tests were excluded if there was a known vanished twin *prior to* testing.^13^ Testing was also not indicated when the gestational age was less than 9 weeks, for triplet or higher multiple pregnancies, or if the patient was known to have used a donor egg. Tests were also excluded if the sample arrived at the laboratory more than 8 days after blood collection, if insufficient blood volume (less than 13 ml), an incorrect collection tube was used, or if the sample was damaged, had hemolysis, or DNA degradation.

Results where the test requisition indicated a twin pregnancy were evaluated for aneuploidy according to an algorithm specifically designed for twin pregnancies.^19^, ^20^ All other tests were evaluated according to a standard algorithm for singleton pregnancies. When evidence of an extra haplotype was seen in a sample processed as a singleton pregnancy, results were not released as a formal written report until after an attempt to contact the referring clinical provider to confirm the pregnancy was singleton. For results where this post‐test checking established the presence of viable twins, the results were reanalyzed using the SNP‐based twin algorithm. Therefore, the only results with extra haplotypes included in this study were those in which the extra alleles were not attributable to a known viable twin at the time of reporting. Analysis was limited to tests with sufficient fetal DNA for aneuploidy risk assessment (>2.8% fetal fraction); cases identified as high risk for triploidy based on a fetal fraction‐based risk assessment (FFBR) were excluded.^21^

Results showing the unexplained presence of an extra haplotype were reported as “consistent with vanishing twin, unrecognized multiple gestation, or fetal triploidy,” and these results were the subject of this study. Pregnancy outcome information was based on either unsolicited information provided by providers or through outreach to referring provider's offices by facsimile, telephone, or both. Follow‐up was performed after the patient's expected date of delivery. Pregnancy outcomes were classified as: confirmed triploidy; suspected triploidy; confirmed vanished twin; suspected vanished twin; pregnancy loss; normal singleton; normal viable twin, or ‘other explanations or multiple factors (see Table 1). Cases of confirmed triplody were based on cytogenetic or cytogenomic testing performed by independent laboratories. For these cases, we also requested information on the parental origin of the extra set of chromosomes, based on molecular genetic analysis and/or evidence of molar changes in the placenta by pathologic examination of placental tissue.

**TABLE 1:** Classifications of outcomes in cases with an extra haplotype

Pregnancy outcome	Definition
Confirmed triploidy	Genetic analysis (chorionic villus sample, amniotic fluid cells, or product of conception sample) confirmed triploidy in the fetus
Suspected triploidy	No genetic testing was performed on the fetus/fetal tissue. However, based on ultrasound findings (cystic placenta, IUGR, discordant head to body size), specific fetal anatomic abnormalities, or abnormal maternal serum screening results typical for triploidy
Confirmed vanished twin	Evidence of a second fetus, second gestational sac, or second fetal pole on sonogram that stopped development or never developed
Suspected vanished twin	One sac or fetus identified on ultrasound but there was early first trimester bleeding and/or in vitro fertilization pregnancy where two embryos were transferred
Pregnancy loss	Pregnancy resulted in spontaneous fetal loss or intrauterine fetal demise with normal or no genetic testing on the fetus/fetal tissue
Normal singleton	No evidence of vanished twin or triploidy
Confirmed viable twins	Twin pregnancies not reported to the laboratory at the time of test referral or in a post‐test contact with the referring provider
Other explanations or multiple factors	Complete molar pregnancy, chimera, other chromosome condition, maternal finding, or complex cases with multiple possible reasons for extra alleles in cfDNA

In results with confirmed or suspected triploidy, the measured fetal fractions were reviewed to determine whether digynic and diandric triploidy could be determined. The methods used to determine fetal fraction (FF) in SNP‐based NIPT are based on the relative proportion of polymorphic alleles in cfDNA contributed by the fetus, with a maximum likelihood value computed from informative loci. This calculation focuses on those chromosomes with a low likelihood of aneuploidy. When triploidy is present, the algorithm will be fitting trisomy data to a disomy SNP model. The FF value returned by the algorithm for a singleton pregnancy with triploidy is therefore not an accurate measure of the true proportion of fetal DNA present. Therefore, we refer to the FF value for triploid pregnancies that is calculated from the singleton pregnancy algorithm as ‘algFF’.

Statistical tests were performed in Excel with a *p*‐value <0.05 considered significant. Confidence intervals (CI) were calculated using an on‐line calculator https://measuringu.com/calculators/wald/.

This study was a retrospective outcome analysis and considered to be a component of quality assurance. As a component of quality assurance, the study received an exempt classification by an Investigative Review Board (Ethical and Independent (E&I), Corte Madera, CA; ID 19040‐01).

## 3 RESULTS

In the 515,804 women screened, 1005 (0.19%, 1 in 513) results indicated an extra haploid set of chromosomes. The mean gestational age at the time of testing for the entire population was 94.1 days (13 weeks, 3 days) and for the cases with an extra haplotype it was 89.4 days (12 weeks, 5 days). The mean maternal age for the population screened was 30.9 years and for those with extra haplotypes present, 33.1 years, respectively. Of the 1005 results where an extra haplotype was identified, pregnancy outcome information was available for 773 (76.9%). Table 1 summarizes the major findings in these pregnancies.

The 773 results with follow‐up information included 58 of confirmed triploidy and 26 where triploidy was suspected (total 84) (Table 2). Viewed as a test for triploidy, the predictive value (PV) for confirmed or suspected triploidy was 10.9% (95% CI 8.9%–13.3%). The frequency of confirmed or suspected triploidy was 84/(515,804*0.769) = 0.021% or 1 in 4722 pregnancies screened. Among the 58 confirmed results, 4 were digynic (6.9%), 23 (39.7%) were diandric and 31 (53.4%) unknown. Under the assumption that the results where the extra set of chromosomes was known reflected all results the incidence of detected digynic triploidy (confirmed or suspected) was (4/(4 + 23))*0.021% = 0.003% or 1 in 31,874 results and the incidence of detected diandric triploidy was (23/(4 + 23)*0.021% = 0.018% or 1 in 5543 results.

**TABLE 2:** Obtained pregnancy outcomes in pregnancies showing an extra haplotype in cell‐free DNA

Pregnancy outcome	Number of cases	%
Confirmed triploidy	58	7.5
Suspected triploidy	26	3.4
Pregnancy loss	75	9.7
Confirmed vanished twin	460	59.5
Suspected vanished twin	39	5.0
Normal singleton	77	10.0
Confirmed viable twins	23	3.0
Other explanations or multiple factors	15	1.9
Total	773	100

Of the 4 identified and confirmed digynic triploidy results, the median algFF was 8.0% (range 6.0%–17.7%), and in the 23 identified and confirmed diandric triploidy results, the median algFF was 11.9% (range 4.3%–28.4%). For these cases, the algFFs were not significantly different from each other (*p* = 0.30) with overlap in the range of values for the two types of triploidy.

The most common finding in results with an extra haplotype was suspected or confirmed vanished twins, present in 499 (65.3%) of the 773 results. These results all constituted findings where the presence of a twin demise was not reported to the laboratory prior to testing. As noted above, our laboratory does not offer NIPT for known vanished twin pregnancies due to the increased likelihood for both false‐positive and false negative results.^22^ The observed overall frequency of results with an undocumented vanished twin in the referral population was 499/(547,325*0.769) = 0.119% or 1 in 843 results. An additional 23 (3.0%) of the results with extra haplotypes had viable twins. These results also included results where a known twin pregnancy was not reported to the laboratory at the time of testing or after attempted contact with the ordering physician. A spontaneous fetal loss after NIPT was found in a further 75 (9.7%) cases. These losses were mostly close to the time of testing and the chromosomal constitution of these fetuses were not established.

Other explanations for extra haplotype were established for an additional 15 results. For three of these results, a complete mole was identified. In one complete molar pregnancy, no fetus was visualized by ultrasound. Presumably, the mole had an etiology involving dispermy with the two paternal haplotypes explaining the extra haplotype. In the second case, the complete mole had a 46,XY karyotype (presumably due to dispermy) and there was also a co‐existing viable 46,XX fetus. Therefore, the extra alleles could be attributed to either the fetus or the mole. In the third case, the complete mole (karyotype unknown) co‐existed with a deceased fetus (karyotype unknown) and, most likely, the extra alleles were attributable to the fetus/mole combination. Two results with a single fetus on ultrasound showed presence of both XX and XY cells, consistent with chimerism. In one of these results, genomic analysis of the products of conception confirmed the presence of an extra set of alleles. In three results, the extra set of alleles was explained following a retrospective review of the medical records that indicated a kidney transplant in the mother. In that clinical situation, the pregnancy, maternal and donor are represented in the cfDNA. In another three results the pregnancies were twin pregnancies from donated eggs, and therefore cfDNA contained SNPs from the gestational carrier, as well as the pregnancy. Another result was associated with melanoma and the circulating tumor DNA from this cancer could have been interpreted as an additional maternal haplotype. An additional result was associated with a triplet pregnancy that spontaneously reduced to twins. In another result, a fetal unbalanced reciprocal translocation was found, but this finding was probably serendipitous because extra alleles affected only one small chromosome segment. One additional result was a complex twin pregnancy with a heterotopic pregnancy and an intrauterine demise.

For the remaining 77 (10%) results no explanation for the extra haplotype was determined. Combining all cases with an explanation (triploidy, viable or non‐viable multiple pregnancy, or other clinically relevant finding), the predictive value of observing an extra haplotype was 90%.

## 4 DISCUSSION

The discovery of an extra haplotype is a rare finding in SNP‐based NIPT. We observed an extra haplotype 1 in every 513 results. The association with vanished twins and triploidy was confirmed,^14^ and in addition, we identified a number of other rare etiologies for the finding.

Approximately 11% of cases were attributable to a triploidy. Data from cytogenetic studies of spontaneous fetal loss have indicated that triploidy is one of the most common cytogenetic abnormalities seen,^23^ and therefore, early NIPT should identify a proportion of these pregnancies. We estimated that in the population of pregnancies with a mean gestational age of 13 weeks 3 days, the frequency of diandric triploidy was approximately 1 in 5543 cases, somewhat higher than estimates made using serum and ultrasound screening (Supplemental Table 1). Conversely, the observed frequency of digynic triploidy, 1 in 31,874, was substantially lower than expected from serum and ultrasound screening. Causes for under ascertainment could be the low FF associated with digynic triploid pregnancy, and also the increased difficulty in detecting digynic triploidy, as compared to diandric, due to the identical nature of the extra haplotype and the background maternal cfDNA in the sample. McKanna et al.^21^ reported that digynic triploidy was 90‐fold more common than expected in those referrals that had a ‘no result’ from NIPT, mostly attributable to low FF. This group of referrals were excluded from this study.

In our reporting of results with an extra haplotype, we did not routinely provide information about the parental origin of the additional alleles. This could potentially distinguish between twins and triploidy and also establish whether the triploidy is digynic or diandric. The combinations of SNPs present in maternal plasma in dizygotic twin pregnancies are complex,^15^ and it can be difficult to distinguish dizygotic twin patterns from diandric triploidy. Our observations also showed that the two types of triploidy cannot be distinguished solely on the overall algFF of the pregnancy; we observed overlap in the values for the two types of triploidy. Despite these complexities, we believe that the algorithm used for allele interpretation can potentially be refined to further assist in the early identification of each type of triploidy.

Our data confirmed that the most common explanation for an extra set of alleles is the presence of a vanishing twin, either confirmed or suspected by ultrasound, in approximately 65% of cases. It has been suggested that the occurrence of a demise of one twin later in pregnancy can be associated with an increased risk for preterm birth.^24^ Identification of a vanishing twin pregnancy could explain abnormal maternal serum markers.^25^, ^26^ In rare instances, SNP‐based testing that demonstrates the presence of a vanishing twin could help avoid false assignment of aneuploidy in a seemingly singleton pregnancy. ACOG Practice Bulletin 226 states that in multifetal gestations, if a fetal demise, vanishing twin, or anomaly is identified in one fetus, there is a significant risk of an inaccurate test result if serum‐based aneuploidy screening or cell‐free DNA is used. This information should be reviewed with the patient and diagnostic testing should be offered.^13^ For patients who decline invasive testing, for the surviving twin, nuchal translucency measurement could be used as a screening tool.^27^

In addition to the vanished twin pregnancies, approximately 3% of results showing an extra haplotype were associated with viable twins. These situations occurred when ultrasound missed one of the fetuses, or the laboratory requisition noted singleton instead of twins. In another 9.7% of results, fetal death was noted at the time of an ultrasound exam, usually performed because of the abnormal results. Altogether, in 75%–80% of the results, an ultrasound is expected to provide an explanation for the extra haplotype result. After an ultrasound exam to evaluate for viable twins, vanishing twins, or fetal death, the predictive value for the testing for triploidy could approach 58/(58 + 77) = 43%. For those results whose ultrasound findings are consistent with triploidy, the predictive value will be even higher.^28^ In our study, only 10% of results with an extra haplotype remained unexplained. Some of these cases could also be attributable to vanished twins because cfDNA can be detectable weeks after a demised co‐twin sac can be identified with ultrasound.^29^

The range of outcomes observed in this group of results suggest a clinical management for women receiving a SNP‐based NIPT result with an extra haplotype. A follow‐up ultrasound should determine whether the cause of the abnormal result was a vanished twin, viable twins, or pregnancy loss. The ultrasound should evaluate for the classic fetal and placental abnormalities characteristic of triploidy, recognizing that not all triploidy are detectable by early ultrasound. The clinical records should be reviewed for history of organ transplant, assisted reproduction with a donor embryo, or a known cancer diagnosis. If performed, maternal serum marker results should be reviewed as well.^11^, ^12^ For definitive prenatal diagnosis of triploidy, an invasive test is indicated. For cases with abnormal ultrasound findings, diagnostic testing can be performed.

A strength of our study is that it is based on a large number of results from a single laboratory. A limitation is the incomplete follow‐up. Results with follow‐up with microarray analysis could miss low level mosaicism, and those with follow‐up karyotype could miss same‐sex chimerism. Also, some women could have had undetected cancer, however, based on our data, this explanation appears to be infrequent. We speculate that, relative to cases with follow‐up, cases with no follow‐up include a higher proportion of pregnancies experiencing loss (without chromosome analysis), and some of these could be attributable to triploidy. Estimates for the triploidy predictive value are therefore expected to be conservative. Our study would also undercount digynic triploidy, where very low FF often precludes any type of NIPT result.^21^ With improvements in testing protocols that allow more reliable interpretation at lower FF, better detection of digynic triploidy can be anticipated.^30^

In summary, detection of triploidy and other conditions associated with an extra haplotype is a secondary benefit of prenatal screening using a SNP‐based NIPT. In conjunction with ultrasound, this testing allows early identification of a small set of high‐risk pregnancies, some of which are also associated with risk to maternal health.

## CONFLICT OF INTEREST

Valerie Kantor, Russ Jelsema, Wenbo Xu, Wendy DiNonno, Kathryn Young and Zachary Demko are employees of Natera, Inc., with stocks or options to own stock in the company. Peter Benn is a consultant to Natera and holds stock options.

## Supporting information

Supplementary Material 1

## Data Availability

The data that support the findings of this study are available from Dr. Z. Demko (zdemko@natera.com) upon reasonable request.
