# Supplementary material for: Non‐invasive prenatal screening for fetal triploidy using single nucleotide polymorphism‐based testing: Differential diagnosis and clinical management in cases showing an extra haplotype
Source: Prenat Diagn. 2022 May 21;42(8):994–9. doi: 10.1002/pd.6169 (PMC9539994; doi:10.1002/pd.6169)
Supplement: Supplementary file 1 — Supplementary Material 1 [file PD-42-994-s001.docx]

Supplemental Table 1. Literature-based estimates of the prevalence of triploidy.

| Trimester | Study | Ascertainment Test (a) | Total population | Digynic Triploidy | Diandric Triploidy | Unspecified Triploidy | All Triploidy |
| --- | --- | --- | --- | --- | --- | --- | --- |
|  |  |  |  |  |  |  |  |
| First | Janiaux et al. 1997^1^ | U/S & Combined | 58,862 | nk | nk | 18 | 18 |
|  | Engelbrechtsen et al. 2003^2^ | Combined | 198,427 | 10 | 3 |  | 30 |
|  | Yaron et al. 2004^3^ | Combined | 12,322 | 6 | 2 | 0 | 8 |
|  | Spencer et al. 2000^4^ | Combined | nk | 10 | 15 | 0 | 25 |
|  | Barken et al. 2008^5^ | Combined | nk | 1 | 4 | 0 | 5 |
|  |  |  |  |  |  |  |  |
| Second | Huang et al. 2005^6^ | Double, Triple & Quadruple | 599,934 | nk | nk | 22 | 22 |

1. U/S ultrasound; Combined Test, nuchal translucency and human chorionic gonadotropin (hCG) and pregnancy associated plasma protein-A (PAPP_A) serum tests; Double, alpha-fetoprotein (AFP) and hCG; Triple, AFP plus hCG plus unconjugated estriol (uE3); Quadruple, AFP, gCG, uE3 plus inhibin-A; nk not known

Based on the combined studies of Janiaux et al., 1997;^1^ Engelbrechtsen et al., 2003;^2^ and Yaron et al., 2004^3^ the incidence of triploidy in the first trimester was 1 in 4,814 pregnancies. Based on all first trimester studies, 27/51 (52.9%) are digynic and 24/51 (47.1%) are diandric. Therefore, the approximate incidence of digynic triplody is 1 in 9,100 and for diandric triploidy it is 1 in 10,221.

1. Jauniaux E, Brown R, Snijders RJ, Noble P, Nicolaides KH. Early prenatal diagnosis of triploidy. Am J Obstet Gynecol. 1997 Mar;176(3):550-4.
2. Engelbrechtsen L, Brøndum-Nielsen K, Ekelund C, Tabor A, Skibsted L; Danish Fetal Medicine Study Group. Detection of triploidy at 11-14 weeks' gestation: a cohort study of 198 000 pregnant women. Ultrasound Obstet Gynecol. 2013 Nov;42(5):530-5.
3. Yaron Y, Ochshorn Y, Tsabari S, Shira AB. First-trimester nuchal translucency and maternal serum free beta-hCG and PAPP-A can detect triploidy and determine the parental origin. Prenat Diagn. 2004 Jun;24(6):445-50.
4. Spencer K, Liao AW, Skentou H, Cicero S, Nicolaides KH. Screening for triploidy by fetal nuchal translucency and maternal serum free beta-hCG and PAPP-A at 10-14 weeks of gestation. Prenat Diagn. 2000 Jun;20(6):495-9.
5. Barken SS, Skibsted L, Jensen LN, Sperling L, Zingenberg H, Brøndum-Nielsen K. Diagnosis and prediction of parental origin of triploidies by fetal nuchal translucency and maternal serum free beta-hCG and PAPP-A at 11-14 weeks of gestation. Acta Obstet Gynecol Scand. 2008;87(9):975-8.
6. Huang T, Alberman E, Wald N, Summers AM. Triploidy identified through second-trimester serum screening. Prenat Diagn. 2005 Mar;25(3):229-33.
